# The Adenosine A_2B_ Receptor Drives Osteoclast-Mediated Bone Resorption in Hypoxic Microenvironments

**DOI:** 10.3390/cells8060624

**Published:** 2019-06-21

**Authors:** Helen J. Knowles

**Affiliations:** Botnar Research Centre, Nuffield Department of Orthopaedics Rheumatology and Musculoskeletal Sciences, University of Oxford, Headington, Oxford OX3 7LD, UK

**Keywords:** osteoclast, bone resorption, hypoxia, adenosine A_2B_ receptor, ATP, hypoxia-inducible factor (HIF), glycolysis, adenosine

## Abstract

Osteoclast-mediated bone destruction is amplified in the hypoxic synovial microenvironment of rheumatoid arthritis (RA). This increased bone resorption is driven by the hypoxia-inducible transcription factor HIF. We identified hypoxic induction of the HIF-regulated adenosine A_2B_ receptor in primary human osteoclasts (mRNA, 3.8-fold increase, *p* < 0.01) and sought to identify the role(s) of purinergic signaling via this receptor in the bone resorption process. Primary human osteoclasts were differentiated from CD14+ monocytes and exposed to hypoxia (2% O_2_) and A_2B_ receptor inhibitors (MRS1754, PSB603). The hypoxic increase in bone resorption was prevented by the inhibition of the A_2B_ receptor, at least partly by the attenuation of glycolytic and mitochondrial metabolism via inhibition of HIF. A_2B_ receptor inhibition also reduced osteoclastogenesis in hypoxia by inhibiting early cell fusion (day 3–4, *p* < 0.05). The A_2B_ receptor is only functional in hypoxic or inflammatory environments when the extracellular concentrations of adenosine (1.6-fold increase, *p* < 0.05) are sufficient to activate the receptor. Inhibition of the A_2B_ receptor under normoxic conditions therefore did not affect any parameter tested. Reciprocal positive regulation of HIF and the A_2B_ receptor in a hypoxic microenvironment thus enhances glycolytic and mitochondrial metabolism in osteoclasts to drive increased bone resorption. A_2B_ receptor inhibition could potentially prevent the pathological osteolysis associated with hypoxic diseases such as rheumatoid arthritis.

## 1. Introduction

Rheumatoid arthritis (RA) is a chronic inflammatory disease characterised by the progressive destruction of bone and articular cartilage. Bone resorption in RA is mediated by multi-nucleated osteoclasts and contributes significantly to disability and poor prognosis [1,2,3]. RA osteoclasts form by the fusion of circulating CD14+ mononuclear precursors or synovial macrophages in the presence of macrophage colony-stimulating factor (M-CSF) and receptor activator for nuclear factor κB ligand (RANKL) [4,5]. Their bone resorption capacity is increased in the hypoxic microenvironment of the rheumatoid joint in a manner dependent on the hypoxia-inducible factor (HIF) transcription factor [6,7].

Increasing attention has been paid to the role of purinergic signaling, a form of extracellular signaling mediated by purine nucleotides and nucleosides, in osteoclast differentiation and function. This has mainly focused on the effect of extracellular ATP to increase the formation and activity of osteoclasts, acting predominantly via the P2Y_1_, P2Y_12_ and P2X_7_ receptors [8]. There is more uncertainty regarding the role of extracellular adenosine, which has variously been reported to stimulate [9,10,11,12], inhibit [13,14] or have no effect on [15,16] osteoclastogenesis and bone resorption. Adenosine stimulates the P1 receptor family (A_1_, A_2A_, A_2B_, A_3_), and the majority of studies have focused on the A_1_ and A_2A_ receptors.

Extracellular adenosine is formed by hydrolysis of extracellular ATP and ADP by ectonucleotidases located in the plasma membrane. ATP release into the extracellular space is increased under conditions of hypoxia or inflammation, via increased vesicular exocytosis from osteoblasts [17] for example, resulting in greatly increased extracellular concentrations of adenosine in hypoxia [18]. Our previous study identified the adenosine A_2B_ receptor (A2BR, ADORA2b) as hypoxia-inducible in primary human osteoclasts [19]. The A_2B_ receptor is hypoxia-inducible in many tissues but is also only activated in hypoxia; because of its low affinity for adenosine (EC_50_ = 24 μM), it is only under hypoxic or inflammatory conditions that sufficient extracellular adenosine is available to activate the A_2B_ receptor [20].

RA is both inflammatory and hypoxic, and therefore adenosine levels would be expected to be elevated in RA patients. Adenosine is very unstable, however, and is hydrolysed by the adenosine deaminase present in synovial fluid, making its actual levels hard to measure clinically, although elevation of adenosine has been observed in murine models of RA [21,22]. High levels of adenosine abolish methotrexate (MTX)-induced suppression of osteoclastogenesis and inflammatory bone destruction in adjuvant-induced arthritis, via a mechanism mediated specifically by the A_2B_ receptor [21]. Clinically, A_2B_ receptor expression is reported within both rheumatoid synovium [23] and rheumatoid nodules [24]. This suggests that elevated concentrations of adenosine in RA may drive resistance to treatments such as MTX via stimulation of osteoclast formation and function.

This work aims to determine whether the A_2B_ receptor mediates the hypoxia-induced increase of osteoclast-mediated bone destruction. It describes how A_2B_ receptor inhibitors specifically reduce hypoxic bone resorption in osteoclasts, which is driven by a hypoxia-induced increase in A_2B_ receptor expression as well as its activation by accumulation of extracellular adenosine. In turn, A_2B_ receptor activation further stabilises HIF-1α to promote bone resorption via an increase in cellular metabolic rate.

## 2. Materials and Methods

### 2.1. Materials and Ethics

Reagents were obtained as follows: human M-CSF (R&D Systems, Abingdon, UK), human soluble RANKL (Peprotech, London UK), MRS1754 and PSB603 (Tocris / BioTechne, Abingdon, UK). Elephant dentine was obtained from HM Revenue & Customs, Heathrow Airport, UK. Unless stated, other reagents were from Sigma-Aldrich (Gillingham, UK). The study was conducted in accordance with the Declaration of Helsinki; the use of leucocyte cones for osteoclast differentiation was approved by the London–Fulham Research Ethics Committee (11/H0711/7).

### 2.2. Osteoclast Differentiation and Cell Culture

CD14+ monocytes were positively selected from the peripheral blood mononuclear cell component of leucocyte cones (NHS Blood and Transplant, UK) using magnetic CD14+ microbeads (Miltenyi Biotech, Surrey, UK). Monocytes were seeded at 1–1.25 × 10^6^ cells/ml onto dentine discs or plastic dishes in α-MEM (without ribonucleosides/deoxyribonucleosides) containing 10% heat-inactivated foetal bovine serum (FBS), 2 mM L-glutamine, 50 IU/ml penicillin and 50 μg/ml streptomycin sulphate. Osteoclastogenesis was induced by treatment with 25 ng/ml M-CSF and 30 ng/ml RANKL every 3–4 days for 9 days. Monocytes were maintained in 25 ng/ml M-CSF. Primary human osteoblasts were purchased from Sigma-Aldrich. Hypoxic exposure was conducted at 2% O_2_, 5% CO_2_, balance N_2_ in a MiniGalaxy incubator (RS Biotech, Irvine, UK).

### 2.3. Osteoclast Formation and Activity Assays

Tartrate-resistant acid phosphatase (TRAP) staining of formalin-fixed cells used naphthol AS-BI phosphate as a substrate, with reaction of the product with Fast Violet B salt. Multi-nucleated cells containing three or more nuclei were considered osteoclasts. Vitronectin receptor (VNR) was detected by immunocytochemistry for CD51/61 (clone 23C6, 1:400; Bio-Rad, Oxford, UK). Resorption tracks produced by mature osteoclasts on dentine discs were visualised by staining with 0.5% toluidine blue following the removal of cells by sonication. Dentines were photographed, resorption tracks highlighted, and the resorbed area quantified using ImageJ (LOCI, Madison, Wisconsin, USA).

### 2.4. Transfection with siRNA and Luciferase Assays

Osteoclasts were transfected with 50 nM siRNA targeting *HIF1α*, *HIF2A* or a *HIF1α* scrambled control using RNAiMAX (Invitrogen, Paisley, UK). Duplexes were removed after 4 h, and osteoclasts were cultured for a further 48 h prior to assay. Cells were transfected with a phosphoglycerate kinase (PGK) hypoxia response element (HRE)–firefly luciferase plasmid ([25]; gifted by Professor AL Harris, University of Oxford, UK) or a pGL4-Per2–luciferase plasmid (containing a 419 bp region of the Per2 promoter around the E-box; gifted by Professor QJ Meng, University of Manchester, UK) as well as a pHRG–TK Renilla luciferase control plasmid (Promega, Southampton, UK) using Lipofectamine 2000 (Invitrogen). Luminescence was assayed after 24 h using the Dual-Luciferase Reporter Assay System (Promega), with firefly luciferase normalized to the Renilla transfection control. To account for potential cyclicity, Per2-luciferase experiments were conducted at the same times of day as the resorption experiments to which they corresponded, cumulative luciferase indicating the overall Per2 promoter activity during this 24 h period.

### 2.5. Realtime PCR

RNA was extracted in TRI reagent (Direct-Zol RNA Miniprep kit; Zymo Research, Irvine, CA, USA) and reverse-transcribed, and quantitative PCR was performed using Fast SYBR Green Master Mix in a Viia7 Real-Time PCR system (Applied Biosystems, Warrington, UK). Human primers were either pre-validated Quantitect primers (Qiagen, Manchester, UK) against *ACTB* (Hs_ACTB_2_SG), A_2B_ receptor-coding sequence (Hs_ADORA2B_1_SG), *HIF1A* (Hs_HIF1A_1_SG) and *HIF2A* (Hs_EPAS1_1_SG) or designed in-house against *CD39* (S: 5’-TTCTCTCCCTCCTTCTGCAA-3’, AS 5’-ATGGCCACTGTGAAAAGGAC-3’), *CD73* (S: 5’-CGCAACAATGGCACAATTAC-3’, AS 5’-CTCGACACTTGGTGCAAAGA) and *PER2* (S: 5’-CGCCCTTTCATCCACATCCT-3’, AS: 5’-AATCCGCTACCACCCCTTCC-3’). Relative quantification (comparative CT) of target gene mRNA was normalised to β-actin (*ACTB*) mRNA.

### 2.6. Western Blotting

Cells were homogenized in HIF lysis buffer (6.2 M urea, 10% glycerol, 5 mM dithiothreitol, 1% sodium dodecyl sulphate, protease inhibitors) or phospho-lysis buffer (1 mM EDTA, 1 mM phenylmethylsulphonyl fluoride (PMSF), 1 mM Na_3_VO_4_, 1 mM NaF in PBS). Gels were evenly loaded with 40–60 μg protein/lane, and molecular weights were compared against the full-range Rainbow molecular weight marker (Fisher Scientific, Loughborough, UK). Primary antibodies were against the A_2B_ receptor (ab1589P, Abcam, Cambridge, UK), HIF-1α (clone 54, 1:1000; BD Biosciences, Oxford, UK), HIF-2α (ep190b, Novus Biologicals, Abingdon, UK), GLUT1 (ab14683, 1:2500; Abcam, Cambridge, UK), NFκB p65 and phospho NFκB p65 (C22B4 and 93H1, Cell Signalling Technology, Leiden, Netherlands) and β-tubulin (clone TUB2.1, 1:2500). Densitometric quantification of Western blots was performed in ImageJ (LOCI, Madison, Wisconsin, USA), normalizing experimental bands to the corresponding β-tubulin control.

### 2.7. Metabolic Assays

Glucose, adenosine and ATP were measured in the media using the Glucose (GO) Assay Kit, the Adenosine Assay Kit (Abcam, Cambridge, UK) and the ATP Determination Kit (ThermoFisher, Loughborough, UK). Lactate was assayed in heat-inactivated medium by the increase in absorbance (340 nm) as NAD+ was converted to NADH in the presence of 0.32 M glycine, 0.32 M hydrazine, 9.6 mM NAD+ and 3 U/ml lactate dehydrogenase. Mitochondrial dehydrogenase activity within the electron transport chain was assessed by adding Alamar blue (BioRad, Kidlington, UK) to cells in culture for 4 h. The results were normalized to the number of osteoclasts.

### 2.8. ELISAs

The concentrations of intracellular cyclic AMP (cAMP) and secreted interleukin-6 (IL-6) were measured using the cAMP Parameter and IL-6 Quantikine ELISA kits (BioTechne, Abingdon, UK), according to the manufacturer’s instructions.

### 2.9. Immunostaining

Osteoclasts were fixed in 4% formalin. The A_2B_ receptor was visualized using the rabbit polyclonal H-200 antibody (Santa Cruz Biotechnology, Heidelberg, Germany) and a PBS control and either the VECTASTAIN Elite Universal ABC Kit and DAB (Vector Laboratories, Peterborough, UK) or an AlexaFluor594 fluorescent secondary antibody. For F-actin ring staining, the cells were permeabilised with 0.5% Triton X-100 and stained with FITC-conjugated phalloidin (0.5 μg/mL). 

### 2.10. Statistical Methods

The results were derived from three or more independent experiments. Data are presented as mean ± standard error of mean (SEM) of data transformed to the mean of the control group. Data were analysed using Prism (Graphpad Software, San Diego, CA, USA). Statistical analysis comprised one-way or two-way ANOVA using Dunnett’s or Tukey’s multiple comparison as a post-hoc test. For experiments with only two conditions, a Students’ t-test was applied. The results were considered significant at *p* < 0.05.

## 3. Results

### 3.1. Hypoxic Induction of the A_2B_ Receptor is HIF-Regulated in Osteoclasts

**Figure 1:**
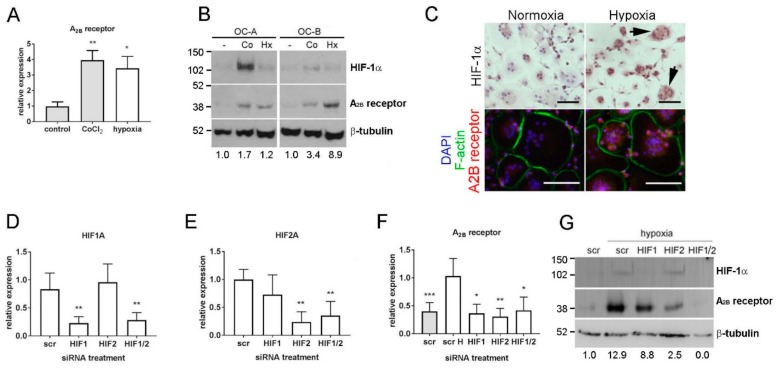
The A_2B_ receptor is regulated by HIF-1α and HIF-2α in osteoclasts. (**A**) A_2B_ receptor mRNA expression is increased by 24 h exposure to CoCl_2_ (100 μM) or hypoxia (2% O_2_). (**B**) Expression of HIF-1α and A_2B_ receptor protein in two independent osteoclast populations (OC-A, OC-B) after 24 h exposure to 100 μM CoCl_2_ (Co) or hypoxia (Hx). Densitometric quantification of the A_2B_ receptor is presented below. (**C**) Immunohistochemistry visualises HIF-1α stabilisation in hypoxic osteoclasts (arrows) and the concomitant increase in A_2B_ receptor expression (red) in multi-nucleated osteoclasts (F-actin ring, green; DAPI, blue). Scale bars = 100 μM. (**D**–**G**) Expression of (**D**) HIF1A, (**E**) HIF-2A and (**F**) A_2B_ receptor mRNA and (**G**) HIF-1α and A_2B_ receptor protein in response to hypoxia (2% O_2_, 24 h; white bars) after HIF-1α, HIF-2α or HIF1α + HIF-2α siRNA, relative to the scrambled (scr) siRNA control. Densitometric quantification of the A_2B_ receptor is presented below; * *p* < 0.05, ** *p* < 0.01, *** *p* < 0.001; *n* = 6.

Our previous microarray analysis of hypoxia-induced genes in human monocyte-derived osteoclasts identified a 2.7-fold hypoxic induction of A_2B_ receptor mRNA (*p* < 0.002) [19] that we confirmed in this study by real-time PCR (Figure 1A; 3.8-fold induction, *p* < 0.01). A_2B_ receptor protein was also induced by hypoxia, with hypoxic conditions verified by stabilisation of HIF-1α (Figure 1B,C). CoCl_2_ induced A_2B_ receptor mRNA and protein, suggesting a role for HIF in its transcriptional regulation. The variation in the magnitude of response to both stimuli is representative of the inter-individual variation characteristic of primary osteoclast preparations. HIF siRNA in osteoclasts achieved 79.7% and 77.1% knock-down of HIF1A and HIF2A mRNAs, respectively (Figure 1D,E) and almost complete ablation of hypoxia-induced HIF-1α protein (Figure 1G; HIF-2α protein is generally undetectable in osteoclasts by Western blot). In hypoxic osteoclasts, both HIF1A and HIF2A siRNA reduced the hypoxic induction of A_2B_ receptor mRNA (Figure 1F) and protein (Figure 1G), indicating that the A_2B_ receptor is regulated by both HIF-1α and HIF-2α in osteoclasts.

### 3.2. The A_2B_ Receptor Drives the Hypoxic Increase in Osteoclast Bone Resorption

**Figure 2:**
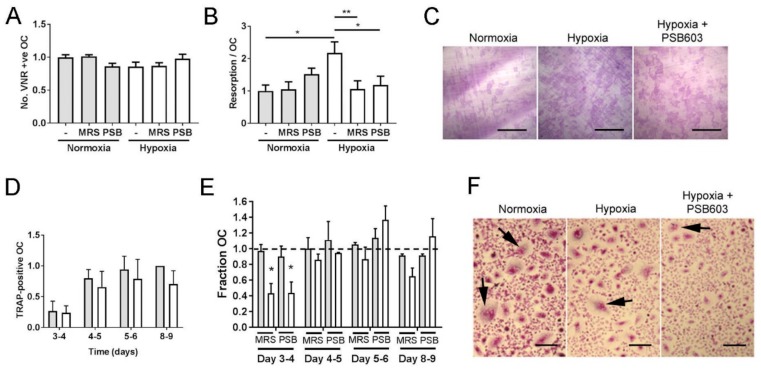
A_2B_ receptor inhibitors reduce osteoclast formation and activity in hypoxic culture. Quantified effect of 24 h exposure of mature human osteoclasts cultured on dentine to MRS1754 (2.5 μM) or PSB603 (10 nM) with respect to (**A**) osteoclast survival (no. VNR-positive osteoclasts present) and (**B**, **C**) the area of dentine resorbed per osteoclast. (*n* = 6). Scale bars = 700 μm. (**D**) Effect of 24 h acute hypoxic exposure (2% O_2_, white bars) on the number of TRAP-positive multi-nucleated osteoclasts formed during differentiation (*n* = 6) and (**E**, **F**) the additional effect of MRS1754 or PSB603 on osteoclastogenesis. Grey bars represent normoxia. (*n* = 3), * *p* < 0.05, ** *p* < 0.01. Arrows indicate multi-nucleated osteoclasts, scale bars = 100 μm..

Hypoxia and HIF moderately inhibit osteoclastogenesis and greatly enhance bone resorption by osteoclasts [6,7]. We therefore investigated whether the A_2B_ receptor might be a HIF-regulated mediator of these processes. The A_2B_ receptor inhibitors MRS1754 and PSB603 were first tested for their effects on the survival and bone resorption capacity of mature osteoclasts. No concentration tested affected osteoclast number (Figure 2A), but both inhibitors were able to prevent the hypoxic increase in osteoclast-mediated bone resorption without affecting the normoxic levels of resorption (Figure 2B,C). Data shown correspond to the lowest concentration of each inhibitor able to prevent the hypoxic increase in bone resorption (2.5 μM MRS1754, 10 nM PSB603); these concentrations were selected for further experiments.

Continuous long-term hypoxia prevents osteoclastogenesis and causes apoptosis of mature osteoclasts [26,27]. We therefore exposed differentiating osteoclasts at different stages of differentiation to a limited 24 h period of hypoxia (Figure 2D), which overall had a moderate suppressive effect on the number of multi-nucleated osteoclasts formed at each stage of differentiation (*p* < 0.05). Exposure to MRS1754 or PSB603 during this 24 h period had no effect under normoxic culture conditions but delayed fusion under hypoxia at the 3–4-day timepoint (Figure 2E,F).

### 3.3. Hypoxic Osteoclasts Secrete ATP to Drive Increased Adenosine Concentrations

**Figure 3:**
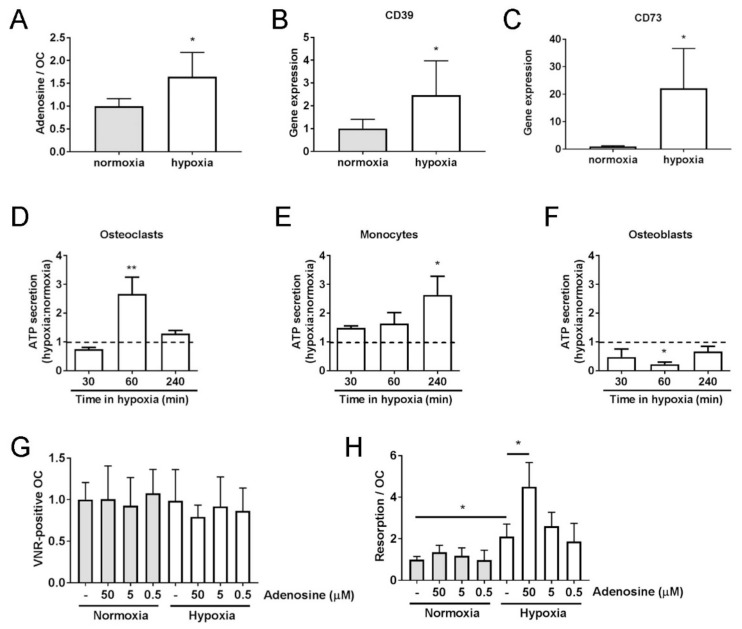
Hypoxic osteoclasts secrete ATP to increase adenosine concentrations. (**A**) A 24 h exposure of mature human osteoclasts to hypoxia (2% O_2_, white bars) increases the concentration of extracellular adenosine (*n* = 4) and (**B**) increases the expression of ectonucleotidase CD39 and (**C**) ectonucleotidase CD73 mRNAs. (*n* = 5). (**D**–**F**) Effect of 30–240 min hypoxia on ATP secretion by (**D**) mature human osteoclasts, (**E**) CD14+ monocytes and (**F**) primary human osteoblasts (*n* = 4). (**G**) Quantified effect of 24 h exposure of mature human osteoclasts to adenosine (0.5–50 μM) with respect to osteoclast survival (number of vitronectin (VNR)-positive osteoclasts present) and (**H**) the area of dentine resorbed per osteoclast. (*n* = 6); * *p* < 0.05, ** *p* < 0.01.

Despite A_2B_ receptor protein being present in both normoxic and hypoxic osteoclasts, inhibition of its activity only affected bone resorption and osteoclastogenesis under hypoxia. This suggests that the concentration of extracellular adenosine, the substrate for the A_2B_ receptor, is sufficiently elevated in in vitro cultured hypoxic osteoclasts to enable activation of the A_2B_ receptor in the absence of any other cell type or stimulus. As hypothesised, extracellular adenosine was elevated 1.6-fold after 24 h in hypoxia (Figure 3A, *p* < 0.05). Extracellular adenosine is formed by the hydrolysis of extracellular ATP by the ectonucleotidases CD39 and CD73, restricting its actions to cells close to the release site. Expression of CD39 and CD73 was also increased in hypoxic osteoclasts (Figure 3B,C). Osteoblasts and osteoclasts constitutively secrete ATP [11,17,28], but only in osteoblasts is this function known to be affected by hypoxia, where acute (<3 min) hypoxic exposure increases ATP release [17]. Acute hypoxic exposure (≤30 min) did not affect osteoclast secretion of ATP; however, longer exposure did elicit this response in osteoclasts (60 min, *p* > 0.01) and their CD14+ mononuclear precursors (Figure 3D–F).

We therefore investigated the effects of adding A_2B_ receptor-activating concentrations of adenosine to osteoclast cultures. Exogenous addition of adenosine only increased osteoclast-mediated bone resorption under hypoxic culture conditions (Figure 3G,H). This suggests that if the effects of exogenously added adenosine are also mediated by the A_2B_ receptor, intracellular signaling after receptor activation must require an additional hypoxia-activated process or pathway.

### 3.4. The A_2B_ Receptor Activates Glycolytic Pathways

Several intracellular signaling pathways downstream of the A_2B_ receptor have potential relevance to osteoclast-mediated bone resorption. These include the interaction of the A_2B_ receptor with the actin cytoskeleton post-stimulation, via its recruitment to the plasma membrane, which initiates the interaction with actin-associated ezrin and actinin-1 [29,30]. Rearrangement of the actin cytoskeleton is vital for osteoclast bone resorption, and although we observed increased F-actin ring formation under hypoxia, inhibition of the A_2B_ receptor did not affect this (Supplementary Figure S1A,B). Activation of the A_2B_ receptor in myeloid cells by exposure to adenosine causes the release of interleukin-6 (IL-6) [31]. IL-6 stimulates both osteoclast formation and activity; however, its production by osteoclasts was not affected by either hypoxia or A_2B_ receptor inhibition (Supplementary Figure S1C).

**Figure 4:**
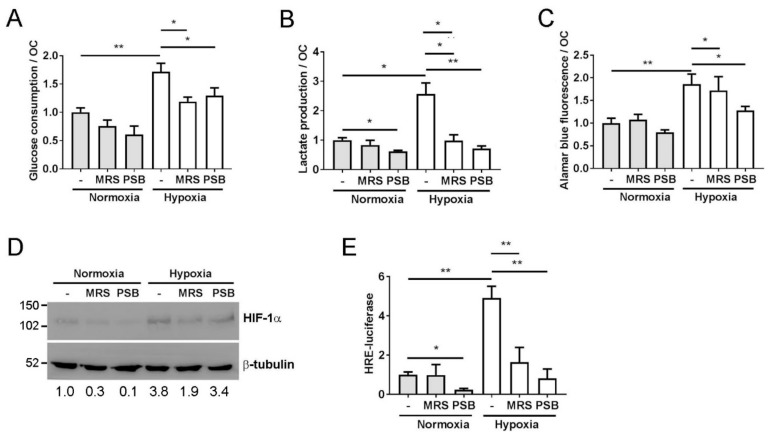
A_2B_ receptor inhibition prevents the HIF-mediated increase in hypoxic glycolysis. Effect of 24 h exposure of mature human osteoclasts to hypoxia (2% O_2_, white bars) and MRS1754 or PSB603 with respect to (**A**) glucose consumption, (**B**) lactate production or (**C**) mitochondrial reductase activity (Alamar blue fluorescence), all per osteoclast (*n* = 8). (**D**) Expression of HIF-1α protein and (**E**) HRE–luciferase activity (*n* = 5) after 24 h exposure of mature human osteoclasts to MRS1754 or PSB603 in either normoxic or hypoxic culture conditions. Densitometry for HIF-1α is presented below the blot; * *p* < 0.05, ** *p* < 0.01.

Activation of the A_2B_ receptor also increases the glycolytic rate via an intracellular feedback loop whereby hypoxic activation of the A_2B_ receptor further activates HIF-1α signaling, which in turn stimulates glycolysis as part of the cellular metabolic adaptation to hypoxia [32,33]. Glycolysis plays a central role in the hypoxic increase in osteoclast-mediated bone resorption [34,35]. Both MRS1754 and PSB603 prevented the hypoxic increase in glucose consumption (Figure 4A) and lactate production (Figure 4B), as well as the osteoclast-specific hypoxic increase in mitochondrial reductase activity (Figure 4C) [34]. Inhibition of the A_2B_ receptor reduced the stabilisation of HIF-1α (Figure 4D) as well as reducing HRE-driven transcription under hypoxia (Figure 4E). One proposed mechanism by which the A_2B_ receptor activates HIF signaling is via the circadian rhythm protein period 2 (Per2). A_2B_ receptor signaling commonly involves adenylyl cyclase and leads to an increase in intracellular cAMP [20], which, in turn, stabilises Per2 and, sequentially, HIF-1α, with consequent effects on glycolysis [33]. However, cAMP and PER2 mRNA levels were reduced in hypoxic osteoclasts, while PER2-driven transcription was increased, although this could potentially be due to the presence of HIF-driven hypoxia-response elements in the Per2 promoter E-box [36]. No effect of A_2B_ receptor inhibition under hypoxia was evident in either case (Supplementary Figure S2).

## 4. Discussion

Within the literature, there is considerable uncertainty regarding the effect of extracellular adenosine on osteoclast formation and activity. Actions of adenosine via the A_1_ receptor generally stimulate osteoclasts [9,10,12], whereas reports of effects via the A_2A_ receptor are divergent [11,13,14]. Exogenous addition of adenosine could potentially affect all four adenosine receptors and result in a combined lack of effect on osteoclast biology [15,16]. Indeed, we also saw no effect of the exogenous addition of adenosine on osteoclast-mediated bone resorption under the normoxic culture conditions in which the above referenced publications were performed.

Exogenous adenosine did increase osteoclast-mediated bone resorption in hypoxic culture, however, suggestive of the requirement for a hypoxia-inducible mediator(s) of this process. The A_2B_ receptor is hypoxia-responsive and is regulated by HIF-1α in human endothelial cells, intestinal and alveolar epithelial cells, dendritic cells and breast cancer cells [37,38,39,40]. There are no previous reports of HIF-2α regulating the A_2B_ receptor, suggesting that its induction by both HIF-1α and HIF-2α in osteoclasts might be cell-type specific. It is unclear why HIF isoform-specific siRNAs comparably reduced A_2B_ receptor mRNA expression, while HIF-2α siRNA more strongly inhibited A_2B_ receptor protein levels. It is possible that the intracellular A_2B_ receptor–HIF feedback loop [32,33] is stronger for HIF-2α than HIF-1α in osteoclasts and could account for this discrepancy; a hypothesis that would be of interest to follow up in the future.

A_2B_ receptor expression in osteoclasts increases during their differentiation from mononuclear precursors [41] and subsequently during hypoxic exposure. The observed intracellular distribution of the A_2B_ receptor in both normoxic and hypoxic osteoclasts was predominantly cytoplasmic as described (www.proteinatlas.org), with a peri-nuclear bias and some nuclear involvement similar to that observed in human sclera fibroblasts and retinal pigment epithelial cells [42,43]. Due to its low affinity for adenosine, the A_2B_ receptor is only activated at the elevated concentrations of extracellular adenosine achieved in hypoxic conditions, despite the protein being present in normoxia. Osteoclast cultures exhibited increased concentrations of ATP and adenosine in the media following acute and extended periods of hypoxic exposure, respectively. Extracellular adenosine is formed by hydrolysis of extracellular ATP and ADP by the ectonucleotidases CD39 and CD73 located in the plasma membrane, whose mRNA levels also increased following hypoxic exposure. The controlled release of ATP from osteoclasts via the P2X_7_ receptor results in the accumulation of extracellular adenosine [11,28], although this process has not been described as hypoxia-regulated. Elevated ATP release by hypoxic osteoblasts via vesicular exocytosis [17] could also contribute to A_2B_ receptor activation in adjacent hypoxic osteoclasts in vivo.

For the reasons above, A_2B_ receptor inhibition with either MRS1754 or PSB603 only suppressed osteoclast fusion and bone resorption in hypoxic culture conditions, when elevated extracellular concentrations of adenosine were sufficient to activate the A_2B_ receptor in the hypoxic cells. Fusogenic actions of extracellular adenosine have been described in both murine and human osteoclast cultures and attributed to the A_1_ or A_2A_ receptors [9,11,12,44]. We did not look at the effects of adenosine on osteoclastogenesis in normoxic culture but found that early fusion in hypoxia was suppressed by the inhibition of the A_2B_ receptor, adding an additional fusogenic role for this receptor to the list. There are potential A_2B_ receptor-independent effects of both inhibitors. MRS1754 is less selective than PSB603 (200-fold versus >17000-fold selectivity) and therefore might partially inhibit other adenosine receptors, although the fact that the effects were only observed in hypoxia would indicate predominant inhibition of the A_2B_ receptor. Despite high selectivity for the A_2B_ receptor, PSB603 increased the rate of oxygen consumption of colorectal cancer cells in an A_2B_ receptor-independent manner [45]. However, this effect was not seen at the low 10 nM concentration used in our study, which also induced hypoxia-dependent and inhibitory effects, again suggestive of a predominantly A_2B_ receptor-driven pathway. 

Prevention of the hypoxic increase in osteoclast-mediated bone resorption by A_2B_ receptor inhibitors suggests that adenosine also stimulates processes involved in bone resorption via the A_2B_ receptor. No effect of A_2B_ receptor inhibitors on bone resorption was seen under normoxic conditions. Previous reports regarding the effects of the A_2B_ receptor on osteoclast activity are opposing. Bone marrow-derived osteoclast formation and bone resorption *ex vivo* was enhanced in cells from A_2B_ receptor knockout mice [46]. However, these results were obtained under normoxic culture conditions in which the A_2B_ receptor should be functionally inactive, suggestive of potential off-target effects in the knock-out animals. The A_2B_ receptor partial agonist BAY60-6583 inhibited precursor fusion and the expression of osteoclast marker genes during differentiation of murine and human bone marrow-derived mononuclear cells into osteoclasts, as well as reduced F-actin ring formation and bone resorption [41,47]. This is harder to reconcile with our data. It is possible that artificial stimulation of the A_2B_ receptor activates alternative intracellular signaling pathways to those activated by physiological stimulation of the receptor, resulting in cellular effects that are not related to the normal cellular functions of the A_2B_ receptor. It has, for example, been noted that in the presence of high levels of adenosine, BAY60-6583 can instead act as an antagonist and block the effects of adenosine at A_2B_ receptors [48].

The adenosine A_2B_ receptor–HIF signaling pathway is one intracellular pathway that would likely not be activated by A_2B_ receptor agonists administered in normoxic culture, due to the requirement for hypoxia to cause inactivation of the HIF-regulating prolyl hydroxylase (PHD) enzymes and subsequent stabilization of HIF-α proteins [7,49]. Adenosine and the A_2B_ receptor amplify HIF signaling under hypoxic conditions, contributing to further stabilisation of HIF protein and activation of HIF-mediated transcription in neuroendocrine enterochromaffin cells of the gut [50], smooth muscle cells [32], oral squamous cell carcinoma cells [51], cardiomyocytes [33] and microglial cells [52], as well as in adenosine deaminase-deficient mice and in vivo models of sickle cell disease [32]. Activation of this signaling pathway leads to an A_2B_ receptor- and HIF-dependent increase in glycolytic metabolism [33,52]. Increased glucose uptake and glycolysis in hypoxic conditions is vital to maintain mitochondrial metabolism and enable osteoclast-mediated bone resorption in these highly energy-dependent cells [34,35]. The fundamental requirement for increased glycolytic and mitochondrial metabolism to enable increased bone resorption in hypoxic osteoclasts explains why adenosine only increases bone resorption activity under hypoxia, when other essential intracellular components of this pathway are also active.

There is evidence that similar pathways are active in osteoclasts in vivo. Plasma adenosine levels are markedly elevated in rats with adjuvant-induced arthritis, a murine model of RA. The time of elevation of adenosine corresponds to the time-point at which the administration of MTX fails to suppress inflammatory bone destruction [21]. In vitro, adenosine abrogates MTX-induced suppression of osteoclastogenesis by suppressing the expression of osteoprotegerin in supporting stromal cells in an A_2B_ receptor-dependent manner [21]. Clinically, the A_2B_ receptor is expressed within both the rheumatoid synovium [23] and rheumatoid nodules [24], suggesting that elevated concentrations of adenosine in RA may drive resistance to treatments such as MTX via multiple mechanisms that stimulate osteoclast formation and function. It is possible that adenosine also drives bone destruction in other diseases. For example, multiple myeloma is a haematological malignancy of terminally differentiated plasma cells that arises within the bone marrow and gives rise to osteolytic bone disease. Elevated extracellular adenosine concentrations in multiple myeloma have been proposed to be indicative of aggressive disease [53], which might in part cause further activation of bone destruction by osteoclasts.

## 5. Conclusions

This study demonstrates that the reciprocal positive regulation of HIF and the A_2B_ receptor in a hypoxic microenvironment enhances glycolytic and mitochondrial metabolism in osteoclasts to promote osteoclast differentiation and drive increased bone resorption. The adenosine A_2B_ receptor–HIF signaling pathway is only active in hypoxic microenvironments, meaning that the inhibition of the A_2B_ receptor only affects these cellular processes under hypoxia. As tissue hypoxia is a disease-specific environment, the A_2B_ receptor could represent a potential therapeutic target, with specificity for hypoxic diseased tissue, which could prevent the pathological osteolysis associated with diseases such as RA.

## Supplementary Materials

The following are available online at https://www.mdpi.com/2073-4409/8/6/624/s1, Figure S1: ADORA2b does not modulate F-actin ring formation or IL-6 secretion in hypoxic osteoclasts. Figure S2: ADORA2b inhibitors do not affect cAMP levels or PER2 expression.
