# Supplementary material for: The Adenosine A2B Receptor Drives Osteoclast-Mediated Bone Resorption in Hypoxic Microenvironments
Source: Cells. 2019 Jun 21;8(6):624. doi: 10.3390/cells8060624 (PMC6628620; doi:10.3390/cells8060624)
Supplement: Supplementary file 1 [file cells-08-00624-s001.pdf]

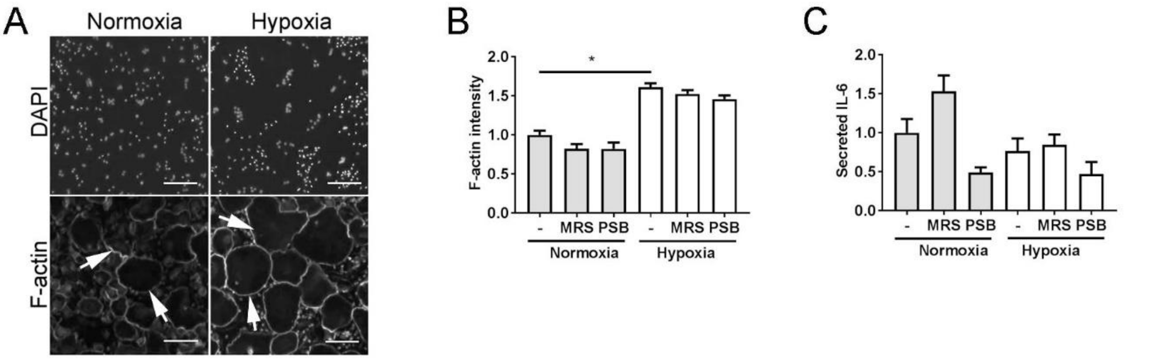

Supplementary Figure 1. The A$_{2B}$ receptor does not modulate F-actin ring formation or IL-6 secretion in hypoxic osteoclasts. (**A**) Fluorescent images and (**B**) quantified fluorescence following F-actin ring staining (arrows) of mature human osteoclasts cultured for 24 h under hypoxia, with MRS1754 or PSB603 (n = 4). Scale bars = 100 µm. (**C**) IL-6 secretion quantified over the same period; *$p < 0.05$, (n = 6).

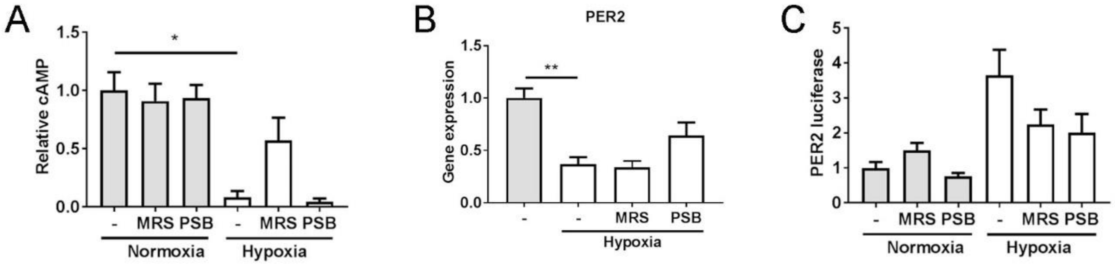

Supplementary Figure 2. A$_{2B}$ receptor inhibitors do not affect cAMP levels or PER2 expression. Effect of hypoxia (2% O_2, 24 h) and MRS1754 or PSB603 on (**A**) intracellular cAMP concentration (n = 4), (**B**) PER2 mRNA expression (n = 6) and (**C**) PER2–luciferase activity in mature human osteoclasts (n = 6). *$p < 0.05$, **$p < 0.01$.
